# Analysis of the Influence of Age, BMI, and WHtR on Body Mass Acceptance, Attitudes, and Motivation towards Body Mass Reduction in Overweight and Obese Caucasian Women

**DOI:** 10.3390/nu11030542

**Published:** 2019-03-03

**Authors:** Maciej Ręgwelski, Ewa Lange, Dominika Głąbska, Dominika Guzek

**Affiliations:** 1 Department of Dietetics, Faculty of Human Nutrition and Consumer Sciences, Warsaw University of Life Sciences (SGGW-WULS), 159c Nowoursynowska Street, 02-776 Warsaw, Poland; maciej_regwelski@sggw.pl (M.R.); dominika_glabska@sggw.pl (D.G.); 2 Department of Organization and Consumption Economics, Faculty of Human Nutrition and Consumer Sciences, Warsaw University of Life Sciences (SGGW-WULS), 159c Nowoursynowska Street, 02-776 Warsaw, Poland; dominika_guzek@sggw.pl

**Keywords:** body mass acceptance, body mass attitudes, body mass reduction, motivation, women, age, body mass index (BMI), waist-to-weight ratio (WHtR), excessive body mass, *BodyMass–DRama* questionnaire, validation

## Abstract

The emotional consequences of excessive body mass, associated with body image and acceptance, have become a global public health challenge as they may decrease the general well-being and hinder weight loss in overweight and obese individuals. Therefore, this study aimed to analyze the influence of age, body mass index (BMI), and waist-to-height ratio (WHtR) on body mass acceptance, attitudes, and motivation toward body mass reduction in overweight and obese Caucasian women with excessive abdominal fat. The previously validated *BodyMass–DRama* (Body Mass–Dietary Restrictions: Acceptance, Motivation, Attitudes) questionnaire was applied in this study. The declared acceptance, attitudes and motivation towards body mass reduction were compared between subgroups based on age (20–40, 40–50, and 50–60 years), BMI (25.0–30.0, 30.0–35.0, and ≥35.0 kg/m^2^), and quartiles of WHtR. The age, BMI and WHtR were stated to be associated with declared acceptance, attitudes, and motivation towards body mass reduction. The different age groups indicated the following as the reasons for excessive body mass: young respondents—low physical activity and consumption of sweets; middle-aged ones—large/irregular meals; aging ones—large/irregular meals and low physical activity (*p* = 0.0161). While describing motivation toward body mass reduction, young respondents indicated the role of a physician or dietitian (*p* = 0.0012) or someone who can control them (*p* = 0.0044), as well as their expectation to be more successful at work after body mass reduction (*p* = 0.0045), while the aging ones indicated appreciation and plaudits from others (*p* = 0.0264) as a motivating factor. Respondents with the highest BMI declared having spending free time actively constricted (*p* = 0.0007); they declared more often than others of feeling exhausted (*p* = 0.0395) or tired all the time (*p* = 0.0445), but less often of feeling full of joy (*p* = 0.0457) or full of energy (*p* <0.0001). Respondents with moderate WHtR declared less often than others that they expect to enjoy socializing (*p* = 0.0376), but more often to be able to have a better vacation after body mass reduction (*p* = 0.0128), while those with the lowest WHtR expected to be more physically active (*p* = 0.0487). Women with the highest WHtR most commonly indicated external pressure from relatives or co-workers as a motivating factor for body mass reduction (*p* = 0.0435). Due to these differences between Caucasian women with excessive body mass, the approach of physicians and dietitians, as well as methods applied to motivate patients, need to be customized.

## 1. Introduction

According to the recent report on the global status of non-communicable diseases published by the World Health Organization (WHO) [1], 38% of adult men and 40% of women are overweight, while 11% of men and 15% of women are obese. As proven in a number of meta-analyses, excessive body mass is associated with a high incidence of health-related consequences, including diabetes [2], hypertension [3], cardiovascular events [4], and chronic kidney disease [5], among others. However, currently, the WHO [6] has also indicated some important social consequences of having excessive body mass called weight bias, which is defined as the negative attitude of others toward individuals, and negative beliefs of others about individuals, with a high body mass [7]. Such a situation may lead to the so-called obesity stigma which is associated with social stigmatization and prejudice resulting in marginalization and inequities against individuals with excessive body mass [8,9].

Weight bias and obesity stigma may not only lead to low self-acceptance of body image, decrease of self-esteem and self-confidence, as well as feelings of worthlessness and loneliness, but also cause general anxiety and depression [6] in overweight and obese individuals. In addition, weight bias and obesity stigma have a negative influence on body mass [10], as they may provoke binge eating [11] and reduce the motivation to lose weight [12]. This negative influence results from a decreased self-acceptance of body mass which leads to overeating in response to negative emotions [13]. Thus, the emotional consequences associated with body mass image and acceptance may decrease the general well-being and hinder weight loss in overweight/obese individuals [14], and are therefore considered as a global public health challenge [15].

To address the indicated public health challenge, understanding the association between age, body mass or body fat distribution, and the emotional consequences of excessive body mass would be valuable. While body mass was so far analyzed as a factor associated with the weight stigma [16,17] and age was also indicated as a factor that may be related [18], there is a lack of studies explaining the complex association between body fat distribution or body stature and emotional consequences of excessive body mass.

Moreover, while the general consequences of obesity are associated both with physical and emotional health impacts [19], the emotional ones may additionally reduce the effectiveness of further body mass reduction [14]. At the same time, so far, they have been commonly neglected [20], so further studies are needed.

Therefore, this study aimed to analyze the influence of age, body mass index (BMI), and waist-to-height ratio (WHtR) on body mass acceptance, attitudes, and motivation toward body mass reduction in overweight and obese Caucasian women with excessive abdominal fat.

## 2. Materials and Methods

### 2.1. Ethical Statement

The study was conducted at the Department of Dietetics, Warsaw University of Life Sciences (WULS-SGGW). The study was conducted according to the guidelines laid down in the Declaration of Helsinki and all procedures involving human subjects were approved by the Ethics Committee of the Faculty of Human Nutrition and Consumer Sciences of the Warsaw University of Life Sciences (No 19/2017). Written informed consent was provided by all participants.

### 2.2. Study Group

The study was conducted on a group of 60 women aged 20–60, who were characterized by excessive body mass, i.e., overweight or obese (BMI ≥ 25.0 kg/m^2^), and had decided to reduce their weight. The sample size was calculated based on the Polish demographical data for the size of the population for 2017 [21], frequency of excessive body mass from the European Health Interview Survey—EHIS, conducted in Poland in 2014 [22], and the frequency of weight stigma in excessive body mass individuals that was estimated as 80% [23], while the confidence level of 95% and margin of error of 10% were taken into account. The study group was recruited by advertisement for 2 weeks in a local Warsaw weekly journal. It was informed that the respondents would participate in a body mass reduction program for 3 months which was free of charge, and that before participation, their acceptance, attitudes, and motivation toward body mass reduction would be assessed by a psychologist. The free of charge body mass reduction program included group meetings with a dietitian (6 meetings in each second week) and a psychologist (6 meetings in each second week) as well as individual meetings with a dietitian (3 meetings in each month) and a psychologist (3 meetings in each month) during the 3-month period. The fact that there was a program organized allowed the assessment of whether individuals had a real intention to reduce body mass through the program, and not just a declarative intention, but was also a kind of compliment for volunteering to participate in the study.

The inclusion criteria were as follows:

- Caucasian origin,
- Overweight or obese—BMI ≥ 25.0 kg/m^2^,
- Excessive abdominal fat distribution—WHtR > 0.5,
- Age 20–60 years,
- Menstruating,
- Declared decision to reduce body mass,
- Informed written consent to participate in the study.

The exclusion criteria were as follows:

- Pregnancy,
- Lactation,
- Allergies and food intolerances,
- Diagnosed with diet-related diseases other than those that are consequences of excessive body mass (hypertension, type 2 diabetes, and dyslipidemia allowed),
- Declined cognitive function.

For proper recruitment of the study group, the physician, psychologist, and dietitian thoroughly verified if the participants fulfilled the inclusion and exclusion criteria during the qualifying procedure. Following recruitment, the assessment of acceptance, attitudes, and motivation toward body mass reduction was conducted among the recruited respondents.

### 2.3. Assessment of BMI and WHtR of the Studied Group

The body mass and height of the respondents were measured to verify if the inclusion criteria were met (in order to obtain a group characterized by BMI ≥25.0 kg/m^2^). The measurements were taken by a professional dietitian, according to the widely accepted and applied rules [24]. The body mass was measured using a calibrated weighing scale (accuracy ± 0.1 kg), and the height was measured using a stadiometer (accuracy ± 0.5 cm). Finally, BMI was calculated based on the Quetelet equation (body mass (kg)/height^2^ (m^2^)) and interpreted according to the criteria of the WHO [25].

Similarly, waist circumference (WC) of the respondents was measured to confirm that the inclusion criteria were met (in order to obtain a group characterized by WHtR >0.5). The measurements were taken by a professional dietitian using a measuring tape (accuracy ± 0.5 cm) in the horizontal plane midway between the lowest ribs and the iliac crest (defined as mid-abdominal WC by the WHO [26] and the International Diabetes Federation (IDF) [27]). Mid-abdominal WC was chosen for the study as it is stated to be a better measurement to define central obesity than the iliac crest WC [28]. Finally, WHtR was calculated from the measured waist and height (waist (cm)/height (cm)) [29].

### 2.4. Stratification into Subgroups by Age, BMI, and WHtR

To analyze the influence of age, the studied group was divided into the following subgroups: young (20–40 years), middle-aged (40–50 years), and aging (50–60 years). In spite of the fact that all the assessed women were non-pregnant, non-lactating, and menstruating, they were divided into different age groups based on the typical course of life: 20–40 years, as a typical maternal age [30]; 40–50 years, as a typical premenopausal age; and 50–60 years, as a typical menopausal age in industrialized countries [31]. As the aim of the study was to assess body mass acceptance, attitudes, and motivation toward body mass reduction, it was assumed that the influence of relatives and the resulting attitude of women may be dependent on their current phase of life.

The characteristics of the studied group for the sub-groups stratified by age are presented in Table 1.

To analyze the influence of BMI, the studied group was divided into the following subgroups according to the criteria of the WHO: overweight (BMI 25.0–30.0 kg/m^2^), moderately obese (BMI 30.0–35.0 kg/m^2^), and significantly obese (BMI ≥35.0 kg/m^2^) [25]. As the aim of the study was to assess body mass acceptance, attitudes, and motivation toward body mass reduction, it was assumed that the influence of relatives and the resulting attitude of women may be dependent on the scale of the problem.

The characteristics of the studied group for the sub-groups stratified by BMI are presented in Table 2.

In spite of the fact that all the assessed women were characterized by excessive abdominal fat distribution, and their WHtR was higher in comparison with the reference value of 0.5 [32], the studied group was divided into the following subgroups based on their WHtR to analyze the influence of WHtR: 1^st^ quartile (Q1, WHtR <0.574), 2^nd^ quartile (Q2, WHtR ϵ <0.574–0.599)), 3^rd^ quartile (Q3, WHtR ϵ <0.599–0.645)) and 4^th^ quartile (Q4, WHtR ≥0.645) (Supplementary Material—Table S1). As the aim of the study was to assess body mass acceptance, attitudes, and motivation toward body mass reduction, it was assumed that the analyzed factors may be dependent on the body shape, even if the general WC was too high.

### 2.5. Assessment of Body Mass Acceptance, Attitudes, and Motivation toward Body Mass Reduction Using BodyMass–DRama Validated Questionnaire (Body Mass–Dietary Restrictions: Acceptance, Motivation, Attitudes)

To assess the body mass acceptance, attitudes, and motivation toward body mass reduction, a dedicated questionnaire, *BodyMass–DRama* (Body Mass–Dietary Restrictions: Acceptance, Motivation, Attitudes), was developed and validated.

The developed *BodyMass–DRama* questionnaire consisted of three parts assessing (1) body mass acceptance (Supplementary Material—Table S2), (2) beliefs, attitudes, and emotions toward body mass (Supplementary Material—Table S3), and (3) motivation toward body mass reduction (Supplementary Material—Table S4). The questionnaire had two questions on body mass acceptance: (1a) to what extent does the respondent accept her body mass and (1b) how does excessive body mass influence the specific aspects of her life (with a list of aspects related to the body mass acceptance). There were two questions on beliefs, attitudes, and emotions toward body mass: (2a) the main reason for excessive body mass of the respondent (open-ended question) and (2b) her emotions during the last 4 weeks (with a list of emotions). There were four questions on motivation toward body mass reduction: (3a) factors that are important for body mass reduction according to the respondent, (3b) factors that motivate her toward body mass reduction (with a list of factors), (3c) the role of family and other relatives as her motivators for body mass reduction, and (3d) expectations associated with body mass reduction (with a list of expectations).

The developed *BodyMass–DRama* questionnaire was validated in an unrelated group of 25 female respondents with excessive body mass who were not undergoing body mass reduction (none of the respondents who participated in the study participated in the validation procedure). The validation process was conducted in two phases to assess the internal consistency and reproducibility of the questionnaire (the same questionnaire was completed twice, while the second one was completed after exactly 6 weeks).

The internal consistency of the *BodyMass–DRama* questionnaire was evaluated by calculating Cronbach’s alpha—the value was 0.82 for the component of body mass acceptance (emotions related to the body mass acceptance), 0.90 for the component of beliefs, attitudes, and emotions (influence of body mass on specific aspects of life), and 0.78 for the component of motivation toward body mass reduction (expectations associated with body mass reduction). The observed Cronbach’s alpha values indicated good-to-excellent internal consistency of the questionnaire [33].

The reproducibility of the *BodyMass–DRama* questionnaire was evaluated using the weighted *κ* statistic and cross-classification method. The values of the weighted *κ* statistic varied from 0.3631 to 0.7807 for the component of body mass acceptance, from 0.2489 to 0.5648 for the component of beliefs, attitudes, and emotions, and from 0.2788 to 0.7788 for the component of motivation toward body mass reduction. The observed weighted *κ* statistic values indicated a fair-to-substantial agreement [34]. At the same time, cross-classification revealed that the share of agreeable answers was 64–92% for the component of body mass acceptance, 44–76% for the component of beliefs, attitudes, and emotions, and 56–100% for the component of motivation toward body mass reduction, confirming the high reproducibility of the questionnaire.

The Paper-and-Pencil Interview was administered by the psychologist for completion of the *BodyMass–DRama* questionnaire, and each respondent was asked to complete the questionnaire on her own without any suggestions. The respondents were instructed to provide the most appropriate answers.

### 2.6. Statistical Analysis

The obtained data were analyzed using:

- Shapiro–Wilk test to analyze the distribution,
- Analysis of variance (ANOVA) to compare sub-groups for parametric distributions,
- Mann–Whitney U test to compare sub-groups for nonparametric distributions,
- Chi^2^ test to compare share of sub-groups,
- Cronbach’s alpha to verify the internal consistency,
- Weighted κ statistic with linear weighting and method of cross-classification to verify the reproducibility.

The statistical analysis was conducted using Statistica, version 8.0 (Statsoft Inc., Tulsa, OK, USA) and Statgraphics Plus for Windows 4.0 (Statgraphics Technologies Inc., The Plains, VA, USA). The level of significance of *p* ≤ 0.05 was chosen.

## 3. Results

### 3.1. Body Mass Acceptance

The body mass acceptance of the studied group for the sub-groups stratified by age is presented in Table 3. It was observed that in the studied group there was no association between age and the factors associated with the body mass acceptance.

The body mass acceptance of the studied group for the sub-groups stratified by BMI is presented in Table 4. From the questionnaire responses, it was observed that there was no association between BMI and the majority of factors related to body mass acceptance in the studied group. Among the respondents, those characterized by BMI ≥35.0 kg/m^2^ (87.0%) more often declared that they have spending their free time activity constricted compared to those who were overweight or moderately obese (*p* = 0.0007).

The responses also showed that there was no association between WHtR and the majority of factors related to body mass acceptance in the studied group (Supplementary material—Table S5). Among the respondents, those characterized by the highest WHtR (Q4, 53.3%) and lowest WHtR (Q1, 46.7%) more often declared that they enjoyed socializing compared to those characterized by moderate WHtR (*p* = 0.0376).

### 3.2. Body Mass Beliefs, Attitudes and Emotions

The body mass beliefs, attitudes, and emotions of the studied group for the sub-groups stratified by age are presented in Table 5. It was also observed that there was no association between age and the emotions during the previous 4 weeks in the studied group. Moreover, the perceived reasons for excessive body mass differed significantly between different age groups (*p* = 0.0161). Respondents aged 20–40 believed that the main reasons for their excessive body mass were lack of physical activity (35%) and consumption of sweets (29%), but they included neither consumption of large meals nor consumption of irregular meals. By contrast, both consumption of large meals and consumption of irregular meals were highlighted as the main reasons of their excessive body mass by respondents aged 40–50 (50% and 25%) and those aged 50–60 (21% and 21%); the aging group also declared lack of physical activity (31%) as a reason for their excessive body mass.

The body mass beliefs, attitudes, and emotions of the studied group for the sub-groups stratified by BMI are presented in Table 6. No association between BMI and the perceived reasons for excessive body mass was observed in the studied group. The emotions during the previous 4 weeks differed significantly between groups with different BMI values. Compared to those characterized by lower BMI, respondents with the highest BMI (≥35.0 kg/m^2^) more often declared that they felt exhausted (75%, *p* = 0.0395) or tired all the time (75%, *p* = 0.0445) but less often declared that they felt full of joy (6%, *p* = 0.0457) or full of energy (18%, *p* < 0.0001).

It was observed that in the studied group there was no association between WHtR and the factors associated with the body mass beliefs, attitudes and emotions (Supplementary Material—Table S6).

### 3.3. Motivation towards Body Mass Reduction

The motivation towards body mass reduction in the studied group for the sub-groups stratified by age is presented in Table 7. It was observed that in the studied group some factors important during body mass reduction and motivating for body mass reduction differed between the age groups. Respondents aged 50–60 less commonly declared the role of physician/dietitian (57.9%) (*p* = 0.0012) and someone who can control them (68.4%) (*p* = 0.0044) than younger ones—those aged 20–40 (100% and 100%, respectively) and 40–50 (91.7% and 95.8%, respectively). However, all of them declared that appreciation and plaudits from others are for them important motivators, that was not observed in younger ones (*p* = 0.0264). At the same time, respondents aged 20–40 declared that they expect being more successful at work after body mass reduction more often (47.1%) than aging ones (*p* = 0.0045).

The motivation towards body mass reduction in the studied group for the sub-groups stratified by BMI is presented in Table 8. It was observed that in the studied group there was no association between BMI and the factors associated with the motivation towards body mass reduction.

The responses also revealed that factors considered important for body mass reduction and as motivating for body mass reduction differed between the WHtR subgroups (Supplementary Material—Table S7). Women characterized by the highest WHtR most commonly stated external pressure from family/relatives or co-workers as a motivating factor for body mass reduction (Q4, 53.3%) (*p* = 0.0435). In addition, their expectations associated with body mass reduction differed. Women characterized by the lowest WHtR most commonly declared that they expected to be more physically active after weight reduction (Q1, 53.3%; Q2, 66.7%) (*p* = 0.0487), while those with moderate WHtR declared that they expected to be able to have better vacation (Q2, 53.3%) (*p* = 0.0128).

## 4. Discussion

### 4.1. Determinants of Emotional Burden of Excessive Body Mass

The present study showed that age, BMI, and WHtR influenced body mass acceptance, beliefs, attitudes, and emotions toward body mass, as well as motivation toward body mass reduction in a group of overweight and obese Caucasian women with excessive abdominal fat. Such observations may be important to achieve body mass reduction.

Nowadays, female bodies that are characterized by a significantly lower fat share than the physiologically healthy range are perceived as most attractive [35]. As a result, women whose appearance not only is not within the attractiveness criteria, but also who do not have a healthy body mass may be seriously influenced by the general perception of their appearance [36]. The dysfunctional body image may undermine their well-being, decrease their self-esteem, reduce life satisfaction, and lead to symptoms of depression and helplessness. Women with a disturbed body image may also consider losing weight as an important element for achieving success [37]. At the same time, the accuracy of body mass estimation also affects the motivation to lose weight, while dissatisfaction with the body image derived from external pressure is strongly linked to worse psychological adjustment and dysfunctional eating behaviors [38].

Besides the general health risk associated with excessive body mass [39] and the resultant mortality [40], another important aspect to be considered is the rising emotional burden [41], as the consequences of excessive body mass are observed not only in the domain of physical health but also in the domains of mental health, social health, and spiritual health [19]. In general, in the case of overweight and obese women, with increase in BMI the risk of depression also increases, as there is a U-shaped association between BMI and depression risk [42].

Psychological interventions may improve the general well-being, as they have been stated to improve the quality of life, mindfulness, and self-compassion abilities, and decrease self-stigma associated with excessive weight, emotional eating, shame, weight-related experiential avoidance, and self-criticism [43]. Individuals with excessive body mass may benefit especially from psychological interventions targeting self-control problems connected with impulsive eating behaviors [44]. Moreover, multidisciplinary, behavior-based weight-loss interventions facilitate more weight loss than usual care programs [45] and thus are highly recommended. The association between weight loss and improved well-being may result from the fact that reduction in distress caused by stigma may lead to avoidance of unhealthy eating and engagement in physical activity, due to reduced stress-induced pathological behaviors [46] and in consequence, it causes improvement of anthropometrical parameters [47].

Thus, it can be emphasized that weight reduction programs should focus on not only the body mass but also the other factors, including psychological and social ones, associated with the possible weight stigma [48]. However, there are also other factors influencing the association; for example, a recent Polish study showed that economic status also influences the association between bad mood and decisions regarding purchase of food products by women [49].

The present study revealed that based on age, BMI, and WHtR, the body mass acceptance, beliefs, attitudes, and emotions toward body mass, as well as motivation toward body mass reduction may differ among individuals, and hence, the actions taken by physicians or dietitians to reduce body mass should also be customized.

### 4.2. Influence of Age on Body Mass Acceptance, Attitudes, and Motivation toward Body Mass Reduction

In the present study, age was found to influence the beliefs, attitudes, and emotions toward body mass, as well as motivation toward body mass reduction, but not body mass acceptance. This influence was confirmed by a study which demonstrated that age at the onset of obesity has a significant impact on the effectiveness of weight-loss programs [50].

In general, it is stated that young women handle overweight issues mainly with a focus on appearance and not on health [51]. In the present study, there was no significant difference observed between younger and older ones with respect to body mass issues associated with either health or appearance, but the strong focus of younger women on body mass issues was confirmed. It may be associated with the fact that a higher number of young respondents in the present study indicated the role of physician or dietitian and someone who can control them as motivators for body mass reduction, but the older ones indicated other motivating factors for body mass reduction. This corresponds with the barriers forfending body mass reduction mentioned by younger respondents in the study by Robertson et al. [52] including limited time, changes in the environment and living situations, availability of unhealthy foods, high cost of recommended food products, and exercise methods, while older respondents mainly indicated age as a barrier reducing the effectiveness of their efforts.

In addition, in the present study, the perception of physical activity as a factor influencing body mass differed among the respondents. While the youngest and aging respondents mentioned low physical activity as one of the factors contributing to their body mass, the middle-aged group did not. Similarly, data from the National Weight Control Registry of the United States of America stated that successful young weight losers considered physical activity as playing an important role in their weight-loss efforts [53]. On the other hand, among physical activity determinants, physical fitness and stress reduction are associated with higher motivation scores with increasing age [54]. This confirms that the middle-aged respondents may be characterized by a lower motivation toward physical activity compared with younger and older ones.

In the present study, the middle-aged respondents mainly indicated factors associated with the general image of diet—large meals or irregular meals—as reasons for their excessive body mass, but neither low physical activity nor sweets consumption which were mentioned by the younger ones. Consumption of sweets is a nutritional habit associated with the predicted body mass [55], and it is commonly known to have a negative influence [56]. Similarly, physical activity is well known to influence body mass [57]. Thus, both reduction of sweets consumption and increase of physical activity require very specific actions and behavioral changes.

At the same time, consumption of large or irregular meals, highlighted by middle-aged respondents, is not a specific and objective feature, and may be interpreted by respondents as independent and not requiring significant effort or important behavioral changes. This may result from the fact that commonly, after finding a partner who she believes is attracted to her body, a woman becomes less attentive to diet and exercise, and thereby less motivated to lose weight [58]. Such attitude may be attributed to the middle-aged women, in the case of whom family and financial stabilization may lead to lower motivation, decreasing the need to change their habits to reduce body mass.

Such low motivation of middle-aged women may be confirmed by the fact that young women and aging ones indicated some important expectations associated with or motivators for body mass reduction. Younger ones expected to be more successful at work after body mass reduction, due to job strain associated with increased BMI [59] and generally perceived a negative effect of body mass on career trajectories [60], which was also observed in a Polish study [61]. It is not surprising that professional career was not indicated as the most important motivating factor by the aging group, but it is quite surprising that this specific aging group indicated appreciation and plaudits from others as very important motivators. This may result from the changing image of their body [62], associated with the inability to accept the natural changes and a need to regain the lost attractiveness, which is typical of women aged over 50 [63]. Due to the fact that middle-aged women would not observe such changes in body at this stage, and their professional career may be already not very important as for the younger ones, this group may be characterized by a lower motivation toward body mass reduction, so all the motivating actions may be especially important for them.

### 4.3. Influence of BMI and WHtR on Body Mass Acceptance, Attitudes, and Motivation toward Body Mass Reduction

In the present study, BMI was found to influence the body mass acceptance, as well as beliefs, attitudes, and emotions towards body mass, but not motivation toward body mass reduction. WHtR was found to influence body mass acceptance, as well as motivation toward body mass reduction, but not beliefs, attitudes, and emotions toward body mass. Such influence is explained by the general observation that the level of stigma toward others differs significantly based on their body mass [64].

Excessive body mass is linked with a number of emotional consequences, including lower weight self-efficacy and higher dissatisfaction with body image [65], as well as burnout, defined as a feeling of deterioration, progressive exhaustion, depletion of energy, and loss of motivation, which may be correlated with BMI [66]. This was consistent with the observations of the present study, as respondents with the highest BMI declared more often than other groups as feeling exhausted and tired all the time and less often as feeling full of joy or full of energy simultaneously. The respondents with the highest BMI also declared having spending free time actively constricted, but also in another Polish study, diet-related compensatory health beliefs were stated by such respondents [67]. However, such a low level of physical activity may be associated with both the objectively constricted physical activity in the overweight and obese [68] and the potential effect of weight stigma on physical activity [69]. Especially for females, even a low level of weight stigma is a potential barrier for engaging in physical activity, as it has a relationship with motivation to exercise [70]. Moreover, it is stated that weight stigma may be a significant factor explaining the positive relationship between high body mass and low level of physical activity [71].

Similar to BMI, WHtR may be indicated as associated with weight stigma, as both BMI and WC are related to perceived weight discrimination [72]. This corresponds with the results observed in the present study, as women with the highest WHtR in the studied group most commonly indicated external pressure from relatives or co-workers as a motivating factor for body mass reduction. This may be associated with the general belief that social pressure may incite weight loss; however, this view is not proven, as it was recently described that weight stigma has a negative effect and may trigger physiological and behavioral changes linked to increased weight gain [14].

The additional observations of the present study were specific for women with moderate WHtR. These respondents less often than others declared that they enjoyed socializing, but it seems that they have such a need as they more often than others expected to be able to have a better vacation after successful body mass reduction. As all the respondents in the studied group were characterized by excessive abdominal fat distribution (WHtR >0.5), those with moderate WHtR must be interpreted as being characterized by abdominal obesity, which is neither mild nor severe. These observations may have resulted from the fear of gaining weight as respondents with moderate WHtR have already experienced some negative social effects due to their body mass and do not want to face even more serious consequences. This can be explained by the higher levels of anxiety in individuals suffering from social rejection due to pervasive anti-fat attitudes in society, which generate a fear of becoming more overweight or extremely obese [73]. Similarly, it is believed that mildly obese individuals may only feel a slight need to change their behaviors or to lose weight for health reasons, while severely obese ones may feel a higher need to change their health behaviors and may blame themselves for their excessive weight [74].

However, it should be emphasized that the defined social fears of participants with moderate WHtR did not generate their constructive expectations associated with body mass reduction. While the group of respondents with the lowest WHtR mainly expected to be more physically active after their body mass reduction, those with moderate WHtR expected to be able to have a better vacation. Such expectation of having a vacation after body mass reduction may have a negative effect on the possibility of maintaining reduced body mass, as in general body weight gain is observed during vacations and for already overweight or obese individuals the weight gain may be even higher, as well as being a major contributor to annual excess weight gain [75]. Thus, such expectation may not allow body mass to be reduced. On the other hand, the opposite effect may be found for those with expectations of being more physically active after body mass reduction, as physical activity may allow the loss of body mass or the reduced body mass to be maintained, as well as possibly causing other positive health-related effects [76]. Due to the fact that individuals with excessive body mass are prone to reward-related consumption [77] and some of their expectations seem to be more risky, patients must be prepared not only for body mass reduction but also for maintaining the reduced body mass and not losing the obtained benefits of body mass reduction.

In spite of the fact that new interesting observations were indicated, some limitations of the study and further study directions should be mentioned. The study was conducted with a relatively small group of women, so future studies should include more respondents, including not only women but also men. Similarly, other age groups should be studied, including children and adolescents, as well as older population. The studied group included only Caucasian women, so the following analysis should be conducted also for other ethnicities. Moreover, also the indication of other factors influencing body mass acceptance, attitudes, and motivation toward body mass reduction is needed, so further potential factors could be included in the analysis.

## 5. Conclusions

1. During body mass reduction, young women with excessive body mass perceived the need for assistance by a physician, dietitian, or someone who can control them, which may have resulted from the experienced barriers forfending body mass reduction.
2. The middle-aged women with excessive body mass were characterized by a lower motivation toward body mass reduction, due to the fact that they did not consider a professional career as important as the younger ones or were not as disturbed by the changing image of their body as the older ones. As a result, they were less prone to change their behaviors, increase their physical activity, or change their diet significantly.
3. The extremely obese women experienced symptoms of burnout, associated with the perceived having spending free time actively constricted, which may escalate the problem of excessive body mass.
4. The subgroup of women with moderate WHtR and abdominal obesity less often declared that they enjoyed socializing, which may have resulted from their fears associated with social rejection already experienced due to anti-fat attitudes in society and their fear of becoming more overweight or extremely obese.
5. There is a need for preparing women with excessive body mass to maintain the body mass after reduction, as some of their expectations may lead to loss of the obtained benefits; this was most commonly stated for the subgroup of women with moderate WHtR.
6. Due to these differences between Caucasian women with excessive body mass, the approach of physicians and dietitians, as well as the methods applied to motivate patients, must be customized taking into account age, BMI, WHtR, and related emotional burden.

## Figures and Tables

**Table 1:** The characteristics of the studied group for the sub-groups stratified by age.

		Age Groups			*p*-Value
		<20–40 years) (*n* = 17)	<40–50 years) (*n* = 24)	<50–60 years> (*n* = 19)	
Age (years)		31.8 ± 5.7 32 (22–39)	44.3 ± 2.7 44 (40–49)	52.2 ± 2.3 52 (50–60)	Not applicable
BMI (kg/m^2^) ^a^		32.3 ± 4.5 31.2 (26.0–46.2)	31.7 ± 5.0 31.4 (25.0–45.4)	34.8 ± 4.9 34.8 (25.7–43.8)	0.0883 *
WHtR (-) ^b^		0.59 ± 0.04	0.60 ± 0.05	0.60 ± 0.06	0.0314 **
Marital status	Married ^c^	8 (47.1%)	19 (79.2%)	14 (73.7%)	0.0770 ***
	Not married ^d^	9 (52.9%)	5 (20.8%)	5 (26.3%)	
Educational background	Secondary education	3 (17.6%)	6 (25.0%)	5 (26.3%)	0.8029 ***
	Higher education	14 (82.4%)	18 (75.0%)	14 (73.7%)	
Self-assessed financial situation	Very bad/bad	0 (0.0%)	0 (0.0%)	0 (0.0%)	0.8945 ***
	Neither bad, nor good	5 (29.4%)	8 (33.3%)	7 (36.8%)	
	Good/very good	12 (70.6%)	16 (66.7%)	12 (63.2%)	

^a^ BMI—Body Mass Index, calculated based on the Quetelet equation (body mass (kg)/height^2^ (m^2^)) [25]; ^b^ WHtR—waist-to-height ratio calculated by dividing waist by height (waist (cm)/height (cm)) [29]; ^c^ defined as married or living in a marriage-like relationship; ^d^ defined as single, widowed, divorced/separated and not in any relationship; * analyzed using Mann–Whitney U test (due to nonparametric distribution; verified using Shapiro Wilk test for *p* ≤ 0.05); ** analyzed using analysis of variance—ANOVA (due to parametric distribution; verified using Shapiro Wilk test for *p* ≤ 0.05); *** analyzed using chi^2^ test.

**Table 2:** The characteristics of the studied group for the sub-groups stratified by BMI.

		BMI Groups			*p*-Value
		<25.0–30.0 kg/m^2^) (*n* = 16)	<30.0–35.0 kg/m^2^) (*n* = 28)	≥35.0 kg/m^2^ (*n* = 16)	
Age (years)		40.4 ± 9.8	42.8 ± 7.1	46.9 ± 8.9	0.1040 *
BMI (kg/m^2^) ^a^		27.5 ± 1.8	32.1 ± 1.5	39.4 ± 3.4	Not applicable
WHtR (-) ^b^		0.56 ± 0.04	0.60 ± 0.04	0.66 ± 0.06	<0.0001 *
Marital status	Married ^c^	8 (50.0%)	7 (25.0%)	4 (25.0%)	0.1837 **
	Not married ^d^	8 (50.0%)	21 (75.0%)	12 (75.0%)	
Educational background	Secondary education	5 (31.2%)	4 (14.3%)	5 (31.2%)	0.3927 **
	Higher education	11 (68.8%)	24 (85.7%)	11 (68.8%)	
Self-assessed financial situation	Very bad/bad	0 (0.0%)	0 (0.0%)	0 (0.0%)	0.5795 **
	Neither bad, nor good	6 (37.5%)	8 (28.6%)	7 (43.7%)	
	Good/very good	10 (62.5%)	20 (71.4%)	9 (56.3%)	

^a^ BMI—Body Mass Index, calculated based on the Quetelet equation (body mass (kg)/height^2^ (m^2^)) [25]; ^b^ WHtR—waist-to-height ratio calculated by dividing waist by height (waist (cm)/height (cm)) [29]; ^c^ defined as married or living in a marriage-like relationship; ^d^ defined as single, widowed, divorced separated and not in any relationship; * analysed using analysis of variance—ANOVA (due to parametric distribution; verified using Shapiro Wilk test for *p* ≤ 0.05); ** analyzed using chi^2^ test.

**Table 3:** The body mass acceptance of the studied group for the sub-groups stratified by age.

		Age Groups			*p*-Value **
		<20–40 years (*n* = 17)	<40–50 years (*n* = 24)	<50–60 years (*n* = 19)	
Body mass acceptance *	Definitely accept	0 (0.0%)	0 (0.0%)	0 (0.0%)	0.1598
	Rather accept	11 (64.7%)	13 (54.2%)	13 (68.4%)	
	Neither accept, nor not accept	1 (5.9%)	0 (0.0%)	2 (10.5%)	
	Rather not accept	0 (0.0%)	5 (20,8%)	3 (15.8%)	
	Not accept at all	5 (29.4%)	6 (25.0%)	1 (5.3%)	
Aspects related to body mass acceptance *	Feeling not attractive	15 (88.2%)	22 (91.7%)	16 (84.2%)	0.7512
	Having problems with socializing	5 (29.4%)	5 (20.8%)	4 (21.1%)	0.7826
	Not feeling good in the presence of slimmer ones	4 (23.5%)	11 (45.8%)	7 (36.8%)	0.3444
	Being not self-confident	11 (64.7%)	15 (62.5%)	12 (63.2%)	0.9894
	Having problem with shopping for clothes	15 (88.2%)	21 (87.5%)	18 (94.7%)	0.7050
	Not feeling comfortable in clothes which she has to wear	12 (70.6%)	16 (66.7%)	11 (57.9%)	0.7102
	Having spending free time actively constricted	6 (35.3%)	11 (45.8%)	11 (57.9%)	0.3960
	Not enjoying socializing	7 (41.2%)	9 (37.5%)	6 (31.6%)	0.8320

* analyzed using *BodyMass–DRama* questionnaire; ** analyzed using chi^2^ test.

**Table 4:** The body mass acceptance of the studied group for the sub-groups stratified by BMI.

		BMI Groups			*p*-Value **
		<25.0–30.0 kg/m^2^ (*n* = 16)	<30.0–35.0 kg/m^2^) (*n* = 28)	≥35.0 kg/m^2^ (*n* = 16)	
Body mass acceptance *	Definitely accept	0 (0.0%)	0 (0.0%)	0 (0.0%)	0.2740
	Rather accept	8 (50.0%)	18 (64.3%)	11 (68.8%)	
	Neither accept, nor not accept	1 (6.2%)	1 (3.6%)	1 (6.3%)	
	Rather not accept	5 (31.3%)	3 (10,7%)	0 (0.0%)	
	Not accept at all	2 (12.5%)	6 (21.4%)	4 (25.0%)	
Aspects related to body mass acceptance *	Feeling not attractive	15 (93.7%)	25 (89.3%)	14 (87.5%)	0.8282
	Having problems with socializing	4 (25.0%)	7 (25.0%)	3 (18.7%)	0.8798
	Not feeling good in the presence of slimmer ones	7 (43.7%)	11 (39.3%)	4 (25.0%)	0.5050
	Being not self-confident	9 (56.3%)	18 (64.3%)	10 (62.5%)	0.8674
	Having problem with shopping for clothes	14 (87.5%)	24 (85.7%)	16 (100.0%)	0.2922
	Not feeling comfortable in clothes which she has to wear	10 (62.5%)	17 (60.7%)	12 (75.0%)	0.6147
	Having spending free time actively constricted	5 (31.3%)	9 (32.1%)	14 (87.0%)	0.0007
	Not enjoying socializing	7 (43.7%)	7 (25.0%)	8 (50.0%)	0.2007

* analyzed using *BodyMass–DRama* questionnaire; ** analyzed using chi^2^ test.

**Table 5:** The body mass beliefs, attitudes, and emotions of the studied group for the sub-groups stratified by age.

		Age Groups			*p*-Value **
		<20–40 years (*n* = 17)	<40–50 years (*n* = 24)	<50–60 years (*n* = 19)	
Perceived reasons of excessive body mass *	Irregular meals	2 (11.8%)	6 (25.0%)	4 (21.1%)	0.0161
	Sweets	5 (29.3%)	1 (4.2%)	1 (5.3%)	
	Snacking	2 (11.8%)	2 (8.3%)	1 (5.3%)	
	Large meals	1 (5.9%)	12 (50.0%)	4 (21.1%)	
	Lack of physical activity	6 (35.3%)	1 (4.2%)	6 (31.5%)	
	Others	1 (5.9%)	2 (8.3%)	3 (15.7%)	
Own emotions during the last 4 weeks *	Full of joy	8 (47.1%)	7 (29.2%)	3 (15.7%)	0.1230
	Very nervous	8 (47.1%)	9 (37.5%)	9 (47.4%)	0.7578
	Sad with no way to be cheered up	4 (23.5%)	5 (20.8%)	4 (21.1%)	0.9759
	Calm and peaceful	10 (58.8%)	9 (37.5%)	8 (42.1%)	0.7389
	Full of energy	9 (52.9%)	8 (33.3%)	5 (26.3%)	0.2310
	Sad and despondent	5 (29.3%)	6 (25.0%)	6 (31.5%)	0.8871
	Exhausted	6 (35.3%)	10 (41.7%)	13 (68.4%)	0.2018
	Happy and lucky	9 (52.9%)	7 (29.2%)	5 (26.3%)	0.1977
	Tired all the time	4 (23.5%)	7 (29.2%)	11 (57.9%)	0.5701

* analyzed using *BodyMass–DRama* questionnaire; ** analyzed using chi^2^ test.

**Table 6:** The body mass beliefs, attitudes, and emotions of the studied group for the sub-groups stratified by BMI.

		BMI Groups			*p*-Value **
		<25.0–30.0 kg/m^2^) (*n* = 16)	<30.0–35.0 kg/m^2^) (*n* = 28)	≥35.0 kg/m^2^ (*n* = 16)	
Perceived reasons of excessive body mass *	Irregular meals	3 (18.8%)	8 (28.6%)	1 (6.3%)	0.7491
	Sweets	2 (12.5%)	3 (10.7%)	2 (12.5%)	
	Snacking	1 (6.3%)	2 (7.1%)	2 (12.5%)	
	Large meals	6 (37.5%)	5 (17.9%)	6 (37.5%)	
	Lack of physical activity	2 (12.5%)	7 (25.0%)	4 (25.0%)	
	Others	2 (12.5%)	3 (10.7%)	1 (6.3%)	
Own emotions during the last 4 weeks *	Full of joy	7 (43.8%)	10 (35.7%)	1 (6.3%)	0.0457
	Very nervous	5 (31.3%)	11 (39.3%)	10 (62.5%)	0.1089
	Sad with no way to be cheered up	4 (25.0%)	6 (21.4%)	3 (18.8%)	0.9126
	Calm and peaceful	8 (50.0%)	14 (50.0%)	5 (31.3%)	0.4346
	Full of energy	7 (43.8%)	11 (39.3%)	3 (18.8%)	<0.0001
	Sad and despondent	4 (25.0%)	7 (25.0%)	6 (37.5%)	0.5687
	Exhausted	7 (43.8%)	10 (35.7%)	12 (75.0%)	0.0392
	Happy and lucky	5 (31.3%)	13 (46.4%)	4 (25.0%)	0.3184
	Tired all the time	6 (37.5%)	11 (39.3%)	12 (75.0%)	0.0445

* analyzed using *BodyMass–DRama* questionnaire; ** analyzed using chi^2^ test.

**Table 7:** The motivation towards body mass reduction in the studied group for the sub-groups stratified by age.

		Age Groups			*p*-Value **
		<20–40 years (*n* = 17)	<40–50 years (*n* = 24)	<50–60 years (*n* = 19)	
Factors important during body mass reduction *	Willpower	17 (100.0%)	22 (91.7%)	18 (94.7%)	0.4822
	Family/relatives	11 (64.7%)	13 (54.2%)	10 (52.6%)	0.7281
	Girlfriends	3 (17.6%)	4 (16.7%)	5 (26.3%)	0.7050
	Group of people with similar problem	5 (29.4%)	12 (50.0%)	6 (31.6%)	0.3133
	Physician/dietitian	17 (100.0%)	22 (91.7%)	11 (57.9%)	0.0012
	Someone who can control me	17 (100.0%)	23 (95.8%)	13 (68.4%)	0.0044
	Diet	15 (88.2%)	21 (87.5%)	19 (100%)	0.2816
	Supplementation/ medicines	1 (5.9%)	1 (4.2%)	2 (10.5%)	0.7002
Factors motivating for body mass reduction *	Deep inner need	17 (100.0%)	24 (100.0%)	19 (100%)	1.0000
	Appreciation and plaudits from others	15 (88.2%)	17 (70.8%)	19 (100%)	0.0264
	External pressure from family/relatives or co-workers	11 (64.7%)	18 (75.0%)	14 (73.7%)	0.7501
Role of family and other relatives as motivators *		14 (82.4%)	22 (91.7%)	17 (89.5%)	0.6464
Expectations associated with body mass reduction *	Being more social person	9 (52.9%)	9 (37.5%)	4 (21.1%)	0.1394
	Smiling more often	9 (52.9%)	9 (37.5%)	6 (31.6%)	0.4045
	Being healthier	17 (100.0%)	24 (100.0%)	18 (94.7%)	0.3338
	Accepting oneself more	13 (76.5%)	14 (58.3%)	13 (68.4%)	0.4696
	Being more accepted by relatives	3 (17.6%)	7 (29.2%)	1 (5.3%)	0.1317
	Taking more care of one’s body	15 (88.2%)	20 (83.3%)	13 (68.4%)	0.5002
	Being able to dress up as one wants	17 (100.0%)	21 (87.5%)	16 (84.2%)	0.2512
	Being more successful at work	8 (47.1%)	1 (4.2%)	4 (21.1%)	0.0045
	Feeling more physically fit	16 (94.1%)	21 (87.5%)	17 (89.5%)	0.7816
	Being more physically active	10 (58.8%)	11 (45.8%)	5 (26.3%)	0.1379
	Having more interesting life	9 (52.9%)	6 (25.0%)	5 (26.3%)	0.1200
	Being able to have better vacation	7 (41.2%)	3 (12.5%)	4 (21.1%)	0.1668

* analyzed using *BodyMass–DRama* questionnaire; ** analyzed using chi^2^ test.

**Table 8:** The motivation towards body mass reduction in the studied group for the sub-groups stratified by BMI.

		BMI Groups			*p*-Value **
		<25.0–30.0 kg/m^2^) (*n* = 16)	<30.0–35.0 kg/m^2^) (*n* = 28)	≥35.0 kg/m^2^ (*n* = 16)	
Factors important during body mass reduction *	Willpower	15 (93.8%)	27 (96.4%)	15 (93.8%)	0.8933
	Family/relatives	11 (68.8%)	13 (46.4%)	10 (62.5%)	0.3060
	Girlfriends	2 (12.0%)	5 (17.9%)	5 (31.3%)	0.3158
	Group of people with similar problem	7 (43.8%)	9 (32.1%)	7 (43.8%)	0.6534
	Physician/dietitian	14 (87.5%)	24 (85.7%)	12 (75.0%)	0.5728
	Someone who can control me	16 (100.0%)	24 (85.7%)	13 (81.3%)	0.2145
	Diet	14 (87.5%)	24 (85.7%)	16 (100.0%)	0.0864
	Supplementation/medicines	1 (6.3%)	2 (7.1%)	1 (6.3%)	0.9905
Factors motivating for body mass reduction *	Deep inner need	16 (100.0%)	28 (100.0%)	16 (100.0%)	1.0000
	Appreciation and plaudits from others	10 (62.5%)	23 (82.1%)	14 (87.5%)	0.1831
	External pressure from family/relatives or co-workers	10 (62.5%)	21 (75.0%)	12 (75.0%)	0.6367
Role of family and other relatives as motivators *		13 (81.3%)	25 (89.3%)	15 (93.7%)	0.5328
Expectations associated with body mass reduction *	Being more social person	6 (37.5%)	11 (39.3%)	5 (31.3%)	0.8652
	Smiling more often	8 (50.0%)	13 (46.4%)	3 (18.8%)	0.1250
	Being healthier	15 (93.7%)	28 (100.0%)	16 (100.0%)	0.2470
	Accepting oneself more	10 (62.5%)	18 (64.3%)	9 (56.3%)	0.8674
	Being more accepted by relatives	1 (6.3%)	5 (17.9%)	5 (31.3%)	0.6534
	Taking more care of ones body	13 (81.3%)	23 (82.1%)	12 (75.0%)	0.1520
	Being able to dress up as one wants	13 (81.3%)	26 (92.9%)	15 (93.8%)	0.2287
	Being more successful at work	3 (18.7%)	4 (14.3%)	6 (37.5%)	0.1880
	Feeling more physically fit	14 (87.5%)	25 (89.3%)	15 (93.8%)	0.8282
	Being more physically active	9 (56.2%)	11 (39.3%)	6 (37.5%)	0.1656
	Having more interesting life	5 (31.3%)	9 (32.9%)	6 (37.5%)	0.9166
	Being able to have better vacation	2 (12.5%)	6 (21.4%)	6 (37.5%)	0.2344

* analyzed using *BodyMass–DRama* questionnaire; ** analyzed using chi^2^ test.

## Supplementary Materials

The following are available online at https://www.mdpi.com/2072-6643/11/3/542/s1, Table S1: The characteristics of the studied group for the sub-groups stratified by WHtR, Table S2: The *BodyMass-DRama* questionnaire (Body Mass — Dietary Restrictions: Acceptance, Motivation, Attitudes) — component of body mass acceptance, Table S3: The *BodyMass-DRama* questionnaire (Body Mass — Dietary Restrictions: Acceptance, Motivation, Attitudes) — component of body mass beliefs, attitudes and emotions, Table S4: The *BodyMass-DRama* questionnaire (Body Mass — Dietary Restrictions: Acceptance, Motivation, Attitudes) — component of motivation towards body mass reduction, Table S5: The body mass acceptance of the studied group for the sub-groups stratified by WHtR, Table S6: The body mass beliefs, attitudes and emotions of the studied group for the sub-groups stratified by WHtR, Table S7: The motivation towards body mass reduction in the studied group for the sub-groups stratified by WHtR.
