# Supplementary material for: Analysis of the Influence of Age, BMI, and WHtR on Body Mass Acceptance, Attitudes, and Motivation towards Body Mass Reduction in Overweight and Obese Caucasian Women
Source: Nutrients. 2019 Mar 3;11(3):542. doi: 10.3390/nu11030542 (PMC6471912; doi:10.3390/nu11030542)
Supplement: Supplementary file 1 [file nutrients-11-00542-s001.pdf]

nutrients

*Article*

# Analysis the influence of age, BMI and WHtR on body mass acceptance, attitudes and motivation towards body mass reduction in overweight and obese Caucasian women

**Maciej Ręgwelski [1], Ewa Lange [1,*], Dominika Głąbska [1] and Dominika Guzek [2]**

[1] Department of Dietetics, Faculty of Human Nutrition and Consumer Sciences, Warsaw University of Life Sciences (SGGW-WULS), 159c Nowoursynowska Street, 02-776 Warsaw, Poland; maciej_regwelski@sggw.pl (M.R.); dominika_glabska@sggw.pl (D.G.)
[2] Department of Organization and Consumption Economics, Faculty of Human Nutrition and Consumer Sciences, Warsaw University of Life Sciences (SGGW-WULS), 159c Nowoursynowska Street, 02-776 Warsaw, Poland; dominika_guzek@sggw.pl
* Correspondence: ewa_lange@sggw.pl; Tel.: +48-22-593-70-25

## Supplementary Material

**Supplementary Table S1.** The characteristics of the studied group for the sub-groups stratified by WHtR.

| | | WHtR groups | | | | p-Value |
|---|---|---|---|---|---|---|
| | | Q1(n=15) | Q2 (n=15) | Q3 (n=15) | Q4 (n=15) | |
| Age (years) | | 41.0±7.9 43 (27-52) | 40.8±9.7 44 (23-53) | 43.0±7.9 42 (28-54) | 47.7±8.7 50 (22-60) | 0.1010* |
| BMI (kg/m$^2$)[a] | | 30.4±5.3 29.7 (25.0-46.2) | 30.9±3.6 30.5 (25.3-36.6) | 33.7±3.4 33.4 (30.0-43.8) | 36.4±5.2 37.2 (29.1-45.4) | 0.0017* |
| WHtR (-)[b] | | 0.54±0.02 | 0.58±0.01 | 0.60±0.01 | 0.67±0.03 | Not applicable |
| Marital status | Married[c] | 8 (53.3%) | 11 (73.3%) | 10 (66.7%) | 12 (80.0%) | 0.0233** |
| | Not married[d] | 7 (46.7%) | 4 (26.7%) | 5 (35.7%) | 3 (20.0%) | |
| Educational background | Secondary education | 3 (20.0%) | 5 (33.3%) | 1 (6.7%) | 5 (33.3%) | 0.2609** |
| | Higher education | 12 (80.0%) | 10 (66.7%) | 14 (93.3%) | 10 (66.7%) | |
| Self-assessed financial situation | Very bad/ bad | 0 (0.0%) | 0 (0.0%) | 0 (0.0%) | 0 (0.0%) | 0.1116** |
| | Neither bad, nor good | 6 (40.0%) | 4 (26.7%) | 2 (13.3%) | 8 (53.3%) | |
| | Good/ very good | 9 (60.0%) | 11 (73.3%) | 13 (86.7%) | 7 (46.7%) | |

[a] BMI – Body Mass Index, calculated based on the Quetelet equation (body mass (kg)/height$^2$ (m$^2$)) [1]; [b] WHtR - waist-to-height ratio calculated by dividing waist by height (waist (cm)/height(cm)) [2]; [c] defined as married or living in a marriage-like relationship; [d] defined as single, widowed, divorced/ separated and not in any relationship; * analyzed using Mann-Whitney U test (due to nonparametric distribution; verified using Shapiro Wilk test for $p \leq 0.05$); ** analyzed using chi$^2$ test.

1. World Health Organization. The challenge of obesity in the WHO European Region and the strategies for response. Summary. Branca F.; Nikogosian, H.; Lobstein T. (eds.). Denmark 2007.
2. Ashwell, M.; Gunn, P.; Gibson, S. Waist-to-height ratio is a better screening tool than waist circumference and BMI for adult cardiometabolic risk factors: systematic review and meta-analysis. *Obes. Rev.* **2012**, *13(3)*, 275-286. doi: 10.1111/j.1467-789X.2011.00952.x.

**Supplementary Table S2.** The *BodyMass-DRama* questionnaire (Body Mass – Dietary Restrictions: Acceptance, Motivation, Attitudes) – component of body mass acceptance.

| Question | | Answers |
|---|---|---|
| **(1a) To what extent do you accept your body mass?** | | Definitely accept<br>Rather accept<br>Neither accept, nor not accept<br>Rather not accept<br>Not accept at all |
| **(1b) How does excessive body mass influence the specific aspects of your life?*** | I feel not attractive. | Definitely yes/ rather yes**<br>Not at all/ rather not** |
| | I have problems with socializing. | Definitely yes/ rather yes**<br>Not at all/ rather not** |
| | I do not feel good in the presence of slimmer ones. | Definitely yes/ rather yes**<br>Not at all/ rather not** |
| | I am not self-confident. | Definitely yes/ rather yes**<br>Not at all/ rather not** |
| | Shopping for clothes is a problem for me. | Definitely yes/ rather yes**<br>Not at all/ rather not** |
| | I do not feel comfortable in clothes which I have to wear. | Definitely yes/ rather yes**<br>Not at all/ rather not** |
| | I can not spend my free time actively. | Definitely yes/ rather yes**<br>Not at all/ rather not** |
| | I do not enjoy socializing. | Definitely yes/ rather yes**<br>Not at all/ rather not** |

* even number of options in order to obtain answers other than neutral ones;
** answers presented separately and combined for analysis.

**Supplementary Table S3.** The *BodyMass-DRama* questionnaire (Body Mass – Dietary Restrictions: Acceptance, Motivation, Attitudes) – component of body mass beliefs, attitudes and emotions.

| Question | | Answers |
| --- | --- | --- |
| **(2a) What is, in your opinion, the main reason of your excessive body mass?** | | *Open-ended question** |
| **(2b) What were your emotions during the last 4 weeks?**** | I felt full of joy. | All the time/ often***<br>Sometimes/ never*** |
| | I was very nervous.**** | All the time/ often***<br>Sometimes/ never*** |
| | I felt sad with no way to be cheered up.**** | All the time/ often***<br>Sometimes/ never*** |
| | I felt calm and peaceful. | All the time/ often***<br>Sometimes/ never*** |
| | I felt full of energy. | All the time/ often***<br>Sometimes/ never*** |
| | I felt sad and despondent.**** | All the time/ often***<br>Sometimes/ never*** |
| | I felt exhausted.**** | All the time/ often***<br>Sometimes/ never*** |
| | I felt happy and lucky. | All the time/ often***<br>Sometimes/ never*** |
| | I felt tired all the time.**** | All the time/ often***<br>Sometimes/ never*** |

* no options presented;
** even number of options in order to obtain answers other than neutral ones;
*** answers presented separately and combined for analysis;

****** during analysis scores reversed.**

**Supplementary Table S4.** The *BodyMass-DRama* questionnaire (Body Mass – Dietary Restrictions: Acceptance, Motivation, Attitudes) – component of motivation towards body mass reduction

| Question | | Answers |
|---|---|---|
| **(3a) Which factors are for you important for body mass reduction?*** | Willpower | Definitely yes/ rather yes**<br>Definitely no/ rather no** |
| | Family/ relatives | Definitely yes/ rather yes**<br>Definitely no/ rather no** |
| | Girlfriends | Definitely yes/ rather yes**<br>Definitely no/ rather no** |
| | Group of people with similar problem | Definitely yes/ rather yes**<br>Definitely no/ rather no** |
| | Physician/ dietitian | Definitely yes/ rather yes**<br>Definitely no/ rather no** |
| | Someone who can control me | Definitely yes/ rather yes**<br>Definitely no/ rather no** |
| | Diet | Definitely yes/ rather yes**<br>Definitely no/ rather no** |
| | Supplementation/ medicines | Definitely yes/ rather yes**<br>Definitely no/ rather no** |
| **(3b) Which factors motivate you toward body mass reduction?** | Deep inner need | Definitely yes/ rather yes**<br>Neither motivate, nor not motivate<br>Rather not/ not at all** |
| | Appreciation and plaudits from others | Definitely yes/ rather yes**<br>Neither motivate, nor not motivate<br>Rather not/ not at all** |
| | External pressure from family/ relatives or co-workers | Definitely yes/ rather yes**<br>Neither motivate, nor not motivate<br>Rather not/ not at all** |
| **(3c) Are the family and other relatives important motivators for your body mass reduction?** | | Definitely yes/ rather yes**<br>Neither motivate, nor not motivate<br>Rather not/ not at all** |
| **(3d) What are your expectations associated with body mass reduction?*** | I will be more social person | Definitely yes/ rather yes**<br>Definitely no/ rather no** |
| | I will smile more often | Definitely yes/ rather yes**<br>Definitely no/ rather no** |
| | I will be healthier. | Definitely yes/ rather yes**<br>Definitely no/ rather no** |
| | I will accept myself more. | Definitely yes/ rather yes**<br>Definitely no/ rather no** |
| | My relatives will accept me more. | Definitely yes/ rather yes**<br>Definitely no/ rather no** |
| | I will take more care of my body. | Definitely yes/ rather yes**<br>Definitely no/ rather no** |
| | I will be able to dress up as I want. | Definitely yes/ rather yes**<br>Definitely no/ rather no** |
| | I will be more successful at work. | Definitely yes/ rather yes**<br>Definitely no/ rather no** |
| | I will feel more physically fit. | Definitely yes/ rather yes**<br>Definitely no/ rather no** |
| | I will be more physically active. | Definitely yes/ rather yes**<br>Definitely no/ rather no** |
| | My life will be more interesting. | Definitely yes/ rather yes**<br>Definitely no/ rather no** |

| | | |
|---|---|---|
| | I will be able to have better vacation. | Definitely yes/ rather yes**<br>Definitely no/ rather no** |

* even number of options in order to obtain answers other than neutral ones;
** answers presented separately and combined for analysis.

**Supplementary Table S5.** The body mass acceptance of the studied group for the sub-groups stratified by WHtR.

| | | WHtR groups | | | | p-Value** |
|---|---|---|---|---|---|---|
| | | Q1(n=15) | Q2 (n=15) | Q3 (n=15) | Q4 (n=15) | |
| Body mass acceptance* | Definitely accept | 0 (0.0%) | 0 (0.0%) | 0 (0.0%) | 0 (0.0%) | 0.8192 |
| | Rather accept | 7 (46.6%) | 10 (66.7%) | 10 (66.7%) | 10 (66.7%) | |
| | Neither accept, nor not accept | 1 (6.7%) | 0 (0.0%) | 1 (6.7%) | 1 (6.7%) | |
| | Rather not accept | 4 (26.7%) | 2 (13.3%) | 2 (13.3%) | 0 (0.0%) | |
| | Not accept at all | 3 (20.0%) | 3 (20.0%) | 2 (13.3%) | 4 (26.7%) | |
| Aspects related to body mass acceptance* | Feeling not attractive | 14 (93.3%) | 13 (86.7%) | 13 (86.7%) | 13 (86.7%) | 0.7985 |
| | Having problems with socializing | 4 (26.7%) | 6 (40.0%) | 3 (20.0%) | 1 (6.7%) | 0.4690 |
| | Not feeling good in the presence of slimmer ones | 9 (60.0%) | 5 (33.3%) | 4 (26.7%) | 4 (26.7%) | 0.1431 |
| | Being not self-confident | 11 (73.3%) | 10 (66.7%) | 7 (46.7%) | 10 (66.7%) | 0.2926 |
| | Having problem with shopping for clothes | 15 (100.0%) | 12 (80.0%) | 13 (86.7%) | 14 (93.3%) | 0.2070 |
| | Not feeling comfortable in clothes which she has to wear | 10 (66.7%) | 8 (53.3%) | 12 (80.0%) | 9 (60.0%) | 0.3012 |
| | Having spending free time actively constricted | 7 (46.7%) | 7 (46.7%) | 5 (33.3%) | 9 (60.0%) | 0.6946 |
| | Not enjoying socializing | 7 (46.7%) | 3 (20.0%) | 1 (7.1%) | 8 (53.3%) | 0.0376 |

* analyzed using *BodyMass-DRama* questionnaire; ** analyzed using $chi^2$ test.

**Supplementary Table S6.** The body mass beliefs, attitudes and emotions of the studied group for the sub-groups stratified by WHtR.

| | | WHtR groups | | | | p-Value** |
|---|---|---|---|---|---|---|
| | | Q1(n=15) | Q2 (n=15) | Q3 (n=15) | Q4 (n=15) | |
| Perceived reasons of excessive body mass* | Irregular meals | 1 (6.7%) | 5 (33.3%) | 2 (13.3%) | 4 (26.7%) | 0.2798 |
| | Sweets | 3 (20.0%) | 2 (13.3%) | 2 (13.3%) | 0 (0.0%) | |
| | Snacking | 2 (13.3%) | 1 (6.7%) | 1 (6.7%) | 1 (6.7%) | |
| | Large meals | 4 (26.7%) | 3 (20.0%) | 4 (26.7%) | 6 (40.0%) | |
| | Lack of physical activity | 1 (6.7%) | 4 (26.7%) | 4 (26.7%) | 3 (20.0%) | |
| | Others | 5 (33.3%) | 0 (0.0%) | 2 (13.3%) | 1 (6.7%) | |
| Own emotions during the last 4 weeks* | Full of joy | 5 (33.3%) | 6 (40.0%) | 6 (40.0%) | 1 (6.7%) | 0.1609 |
| | Very nervous | 6 (40.0%) | 6 (40.0%) | 5 (33.3%) | 9 (60.0%) | 0.4856 |
| | Sad with no way to be cheered up | 6 (40.0%) | 3 (20.0%) | 0 (0.0%) | 4 (26.7%) | 0.0611 |
| | Calm and peaceful | 6 (40.0%) | 6 (40.0%) | 9 (60.0%) | 6 (40.0%) | 0.6110 |
| | Full of energy | 6 (40.0%) | 6 (40.0%) | 7 (46.7%) | 3 (20.0%) | 0.4306 |
| | Sad and despondent | 5 (33.3%) | 4 (26.7%) | 2 (13.3%) | 6 (40.0%) | 0.4117 |
| | Exhausted | 7 (46.7%) | 10 (67.7%) | 3 (20.0%) | 9 (60.0%) | 0.0532 |
| | Happy and lucky | 5 (33.3%) | 5 (33.3%) | 7 (46.7%) | 4 (26.7%) | 0.7074 |
| | Tired all the time | 6 (40.0%) | 9 (60.0%) | 6 (40.0%) | 8 (53.3%) | 0.6145 |

* analyzed using *BodyMass-DRama* questionnaire; ** analyzed using $chi^2$ test.

**Supplementary Table S7.** The motivation towards body mass reduction in the studied group for the sub-groups stratified by WHtR.

| | | WHtR groups | | | | p-Value** |
|---|---|---|---|---|---|---|
| | | Q1(n=15) | Q2 (n=15) | Q3 (n=15) | Q4 (n=15) | |
| Factors important during body mass reduction* | Willpower | 14 (93.3%) | 13 (86.7%) | 15 (100.0%) | 15 (100.0%) | 0.2770 |
| | Family/ relatives | 7 (46.7%) | 10 (66.7%) | 10 (66.7%) | 7 (46.7%) | 0.4856 |
| | Girlfriends | 1 (6.7%) | 2 (13.3%) | 4 (40.0%) | 3 (20.0%) | 0.1350 |
| | Group of people with similar problem | 6 (40.0%) | 5 (33.3%) | 7 (46.7%) | 5 (33.3%) | 0.8553 |
| | Physician / dietitian | 12 (80.0%) | 13 (86.7%) | 14 (93.3%) | 11 (73.3%) | 0.4936 |
| | Someone who can control me | 14 (93.3%) | 13 (86.7%) | 13 (86.7%) | 13 (86.7%) | 0.9221 |
| | Diet | 13 (86.7%) | 15 (100.0%) | 14 (93.3%) | 14 (93.3%) | 0.5433 |
| | Supplementation/ medicines | 0 (0.0%) | 1 (6.3%) | 1 (6.3%) | 2 (13.3%) | 0.5433 |
| Factors motivating for body mass reduction* | Deep inner need | 15 (100.0%) | 15 (100.0%) | 15 (100.0%) | 15 (100.0%) | 1.0000 |
| | Appreciation and plaudits from others | 12 (80.0%) | 14 (93.3%) | 13 (86.7%) | 12 (80.0%) | 0.6967 |
| | External pressure from family/ relatives or co-workers | 9 (60.0%) | 9 (60.0%) | 10 (66.7%) | 15 (100.0%) | 0. 0435 |
| Role of family and other relatives as motivators* | | 12 (80.0%) | 14 (93.3%) | 14 (93.3%) | 13 (86.7%) | 0.6195 |
| Expectations associated with body mass reduction* | Being more social person | 5 (33.3%) | 7 (46.7%) | 5 (33.3%) | 5 (33.3%) | 0.8348 |
| | Smiling more often | 7 (46.7%) | 7 (46.7%) | 5 (33.3%) | 5 (33.3%) | 0.7744 |
| | Being healthier | 14 (93.3%) | 15 (100.0%) | 15 (100.0%) | 15 (100.0%) | 0.3838 |
| | Accepting oneself more | 10 (66.7%) | 10 (66.7%) | 9 (60.0%) | 11 (73.3%) | 0.8481 |
| | Being more accepted by relatives | 0 (0.0%) | 4 (26.7%) | 4 (26.7%) | 3 (20.0%) | 0.1881 |
| | Taking more care of ones body | 12 (80.0%) | 12 (80.0%) | 15 (100.0%) | 9 (60.0%) | 0.5730 |
| | Being able to dress up as one wants | 13 (86.7%) | 13 (86.7%) | 15 (100.0%) | 13 (86.7%) | 0.5276 |
| | Being more successful at work | 2 (13.3%) | 5 (33.3%) | 3 (20.0%) | 3 (20.0%) | 0.6007 |
| | Feeling more physically fit | 13 (86.7%) | 14 (93.3%) | 13 (86.7%) | 14 (93.3%) | 0.8636 |
| | Being more physically active | 8 (53.3%) | 10 (66.7%) | 3 (20.0%) | 5 (33.3%) | 0.0487 |
| | Having more interesting life | 3 (20.0%) | 7 (46.7%) | 5 (33.3%) | 5 (33.3%) | 0.4936 |
| | Being able to have better vacation | 1 (6.7%) | 8 (53.3%) | 3 (20.0%) | 2 (13.3%) | 0.0128 |

* analyzed using *BodyMass-DRama* questionnaire; ** analyzed using $chi^2$ test.
